# Renal stromal miRNAs are required for normal nephrogenesis and glomerular mesangial survival

**DOI:** 10.14814/phy2.12537

**Published:** 2015-10-05

**Authors:** Yu Leng Phua, Jessica Y S Chu, April K Marrone, Andrew J Bodnar, Sunder Sims-Lucas, Jacqueline Ho

**Affiliations:** 1 Rangos Research Center, School of Medicine, Children’s Hospital of Pittsburgh of UPMC, University of PittsburghPittsburgh, Pennsylvania; 2 Department of Pediatrics, Division of Nephrology, School of Medicine, University of PittsburghPittsburgh, Pennsylvania

**Keywords:** Glomerular mesangium, kidney development, microRNAs, renal stroma

## Abstract

MicroRNAs are small noncoding RNAs that post-transcriptionally regulate mRNA levels. While previous studies have demonstrated that miRNAs are indispensable in the nephron progenitor and ureteric bud lineage, little is understood about stromal miRNAs during kidney development. The renal stroma (marked by expression of *FoxD1*) gives rise to the renal interstitium, a subset of peritubular capillaries, and multiple supportive vascular cell types including pericytes and the glomerular mesangium. In this study, we generated *FoxD1*^*GC*^*;Dicer*^*fl/fl*^ transgenic mice that lack miRNA biogenesis in the *FoxD1* lineage. Loss of Dicer activity resulted in multifaceted renal anomalies including perturbed nephrogenesis, expansion of nephron progenitors, decreased renin-expressing cells, fewer smooth muscle afferent arterioles, and progressive mesangial cell loss in mature glomeruli. Although the initial lineage specification of FoxD1^+^ stroma was not perturbed, both the glomerular mesangium and renal interstitium exhibited ectopic apoptosis, which was associated with increased expression of *Bcl2l11 (Bim)* and p53 effector genes (*Bax*, *Trp53inp1*, *Jun*, *Cdkn1a*, *Mmp2*, and *Arid3a*). Using a combination of high-throughput miRNA profiling of the FoxD1^+^-derived cells and mRNA profiling of differentially expressed transcripts in *FoxD1*^*GC*^*;Dicer*^*fl/fl*^ kidneys, at least 72 miRNA:mRNA target interactions were identified to be suppressive of the apoptotic program. Together, the results support an indispensable role for stromal miRNAs in the regulation of apoptosis during kidney development.

## Introduction

Mammalian kidney formation results from complex and tightly regulated processes involving crosstalk between the ureteric bud and metanephric mesenchyme (reviewed in Little and McMahon 2012). The ureteric bud is induced via signals from the metanephric mesenchyme to give rise to the collecting system of the kidney. In turn, the metanephric mesenchyme is induced by the ureteric bud to form a “cap” of nephron progenitors around each ureteric bud. Wnt9b/*β*-catenin signaling from the ureteric tips causes the surrounding Six2^+^ nephron progenitors to either commit toward epithelial differentiation or promote self-renewal such that the progenitor pool is not prematurely depleted prior to cessation of nephrogenesis (Karner et al. 2011). The cortical renal stroma develops immediately adjacent to the nephron progenitors, and is thought to provide critical signals to nephron progenitors as they undergo self-renewal and differentiate into functional nephrons (reviewed in Cullen-Mcewen et al. 2005). Indeed, mutations that affect the renal stroma are known to result in aberrant nephron differentiation, branching morphogenesis, and subsequent renal hypodysplasia (Hatini et al. 1996; Mendelsohn et al. 1999; Quaggin et al. 1999; Schnabel et al. 2003; Levinson et al. 2005; Das et al. 2013; Hum et al. 2014). Recent work has demonstrated that the distinctive opposing effects of Wnt9b/*β*-catenin signaling on nephron progenitors is modulated in part by the adjacent FoxD1^+^ cortical renal stroma via Fat4/Yap signaling (Das et al. 2013).

Furthermore, the FoxD1^+^ renal stroma also constitutes a progenitor pool for several key renal lineages, such as the renal interstitium, various supportive vascular cells (capillary pericytes, interstitial fibroblasts, afferent arteriole smooth muscle, renin-expressing cells, and glomerular mesangial cells) (Humphreys et al. 2010; Kobayashi et al. 2014) and potentially a small subset of peritubular capillaries (Sims-Lucas et al. 2013). Thus, abnormalities of the renal stroma affect vascular patterning in the kidney (Levinson et al. 2005). Since mesangial cells are required for the formation of normal capillary loops, and function as a dynamic mechanical support in response to changes in intraglomerular pressures, the renal stroma is also required for normal glomerular development (Lindahl et al. 1998; Kikkawa et al. 2003).

While various transgenic mouse models and genome-wide association screens have successfully identified causative genes implicated in congenital abnormalities of the kidney and urinary tract, the role of miRNA post-transcriptional regulation during kidney development remains poorly understood. MicroRNAs (miRNAs) are small, endogenous, noncoding RNAs that post-transcriptionally regulate the mRNA levels (Ebert and Sharp 2012; Ho and Kreidberg 2012). During miRNA biogenesis, Dicer catalyzes the formation of mature miRNA from precursor miRNAs (pre-miRNA). Mature miRNAs function by binding to their target mRNAs, an interaction that is largely dependent on an 8-seed nucleotide sequence (Grimson et al. 2007). Upon complementary binding and miRNA–mRNA duplex formation, RNA-induced silencing complex (RISC) is recruited, leading to repression of mRNA transcripts or inhibition of protein translation (Ho and Kreidberg 2012).

In the context of kidney development and disease, we and others have previously shown that miRNAs are indispensable for the development of the kidney (Harvey et al. 2008; Shi et al. 2008; Sequeira-Lopez et al. 2010; Wei et al. 2010; Ho et al. 2011; Nagalakshmi et al. 2011; Zhdanova et al. 2011; Chu et al. 2014; Marrone et al. 2014; Phua and Ho 2015). Specifically, deletion of *Dicer* within the Six2^+^ nephron progenitors and Hoxb7^+^ ureteric bud lineages resulted in premature nephron progenitor depletion and cystic renal disease, respectively (Ho et al. 2011; Nagalakshmi et al. 2011). Similarly, lack of miRNA post-transcriptional regulation in glomerular podocytes resulted in podocyte loss and onset of chronic kidney disease during adulthood (Harvey et al. 2008; Ho et al. 2008; Shi et al. 2008; Zhdanova et al. 2011). Deletion of *Dicer* in renin-expressing cells is required for the maintenance of the juxtaglomerular apparatus (Sequeira-Lopez et al. 2010). Yet, the functional role of miRNAs in the cortical renal stroma is still undetermined.

In this study, we generated a *FoxD1*^*GC*^*;Dicer*^*fl/fl*^ transgenic mouse model that lacks miRNA biosynthesis in the FoxD1^+^ renal stroma lineage and its cellular derivatives. *FoxD1*^*GC*^*;Dicer*^*fl/fl*^ embryos developed a multifaceted renal phenotype that is characterized by reduced nephron endowment, increased nephron progenitors, reduced smooth muscle in afferent arterioles, and decreased renin-expressing cells. With respect to glomerular development, *FoxD1*^*GC*^*;Dicer*^*fl/fl*^ kidneys exhibited progressive mesangial defects, accompanied by a failure of mesangial cell maintenance and onset of microaneurysms in mature glomeruli. Using a combination of high-throughput miRNA profiling and transcriptome microarray analysis, we curated a candidate list of miRNAs expressed in the renal stroma lineage, along with differentially expressed transcripts in *FoxD1*^*GC*^*;Dicer*^*fl/fl*^ kidneys. In this manner, we identified at least 72 potential dysregulated miRNA:mRNA interactions that are likely to contribute to the underlying etiology of the *FoxD1*^*GC*^*;Dicer*^*fl/fl*^ renal phenotype. Finally, we showed that the phenotypic defects are likely to be due, at least in part, to misregulation of cellular apoptosis via multiple mechanisms – ectopic expression of the proapoptotic protein Bim and elevated levels of downstream p53 effector genes including *Bax*, *Trp53inp1, Jun*, and *Cdkn1a*.

## Materials and Methods

### Mouse strains

The *FoxD1*^*GC*^ mouse line (obtained from the Jackson Laboratory, Strain B6;129S4-*Foxd1*^*tm1(GFP/cre)Amc*^/J) (Humphreys et al. 2010; Kobayashi et al. 2014) expresses an enhanced green fluorescent protein (eGFP)-Cre recombinase fusion protein from the endogenous FoxD1 locus, resulting in expression of the eGFP-Cre protein in the renal stroma and were maintained on a *129-Elite* background. The *Rosa*^*CAG-tdTomato*^ reporter mice express a CAG-promoter–driven red fluorescent protein variant (tdTomato) upon excision of a loxP-flanked stop cassette, and were maintained on a *C57BL/6* background (from Jackson Laboratory, strain B6.Cg-Gt(ROSA)26Sor^tm9(CAG-tdTomato)Hze^/J). These mice were crossed with a conditionally floxed *Dicer* allele (from Jackson Laboratory, B6.Cg-*Dicer1*^*tm1Bdh*^/J) (Harfe et al. 2005), which is required for the production of mature miRNAs, to generate *FoxD1*^*GC*^*;Dicer*^*fl/fl*^ and *FoxD1*^*GC*^*;Dicer*^*fl/fl*^;*tdTomato* embryos. The *Dicer*^*fl/fl*^ animals were maintained on a *C57BL/6J* background. Cre-negative littermates were used as controls. Timed matings were performed and the day on which plugs were observed was considered embryonic day 0.5 (E0.5). Embryonic tissue was genotyped by polymerase chain reaction (PCR) with the following primers as described previously: (1) *FoxD1*^*GC*^ allele, forward primer 5′-GGG AGG ATT GGG AAG ACA AT-3′ and reverse primer 5′-TCT GGT CCA AGA ATC CGA AG-3′, yielding a 450-bp band indicating the presence of Cre recombinase (Kobayashi et al. 2014); and (2) *Dicer*^*fl*^ allele, forward primer 5′-CCT GAC AGT GAC GGT CCA AAG-3′ and reverse primer 5′-CAT GAC TCT TCA ACT CAA ACT-3′, yielding a 350-bp wild-type band and a 400-bp *Dicer*^*fl*^ band (Harfe et al. 2005). *FoxD1*^*GC*^*;tdTomato;Dicer*^*fl/fl*^ and control littermates were identified by tdTomato fluorescence. All animals were housed in the Vivarium at the Rangos Research Center at the Children’s Hospital of Pittsburgh of UPMC, Pittsburgh, Pennsylvania, and all animal experiments were carried out in accordance with the policies of the Institutional Animal Care and Use Committee at the University of Pittsburgh.

### Histopathological and immunofluorescence staining

Dissected embryos and kidneys were imaged on a Leica M165 dissecting microscope. Measurements were performed using ImageJ, and kidney length was normalized to crown–rump length. At least four animals of each genotype across three litters were measured. Samples were fixed in 4% paraformaldehyde overnight at 4°C, processed for embedding in paraffin, and sectioned at 4 *μ*m. For cryosections, fixed kidneys were cryoprotected in 30% sucrose, embedded in Tissue-Tek OCT (Sakura Finetek, Torrance, CA), and sectioned at 8 *μ*m. Glomerular aneurysm counting was performed on three litters of E18.5 control and *FoxD1*^*GC*^*;Dicer*^*fl/fl*^ midsagittal sections. Images of the entire section were acquired from four consecutive serial sections from each sample, and quantitation was performed using ImageJ. The number of glomeruli with aneurysms was normalized to the total number of glomeruli counted and expressed as a relative percentage. The two-tailed Student’s *t*-test was used to determine statistical significance.

Whole-mount immunofluorescence staining was performed on E13.5, 15.5, and 19.5 kidneys and z-stack images acquired on an Olympus Confocal Microscope. Section immunofluorescence was carried out on midsagittal paraffin sections or cryosections. For paraffin sections, slides were first deparaffinized, rehydrated, and antigen retrieved by boiling the slides in 10 mmol/L pH 6.0 sodium citrate buffer. A Mouse on Mouse blocking and biotin/streptavidin blocking kit (Vector Labs, Burlingame, CA) were used for antibodies raised in mouse. Primary antibodies were detected with their respective secondary Alexa Fluor conjugated antibodies (Life Technologies, Waltham, MA), streptavidin (Jackson ImmunoResearch, Westgrove, PA), Tyramide Signal Amplification (TSA) (Life Technologies) or Vectastain Elite ABC Kit-DAB peroxidase substrate (Vector Labs). Primary antibodies were used at 1:100 dilution and include: Six2 (Proteintech #11562-1-AP, Chicago, IL), Forkhead box D1 (FoxD1) (Santa Cruz #sc-47585, Dallas, TX), Calbindin (Sigma #C9848, St. Louis, MO), annexin A2 (Anxa2) (Cell Signaling #8235, Danvers, MA), Neural cell adhesion molecule (Ncam) (Sigma #C9672), Tenascin C (Millipore #AB19011, Billerica, MA), Yes-associated protein 1 (Yap) (Cell Signaling #4912), phospho-Yap (pYap) (Cell Signaling #4911), Desmin (Dako #M076029-2, Carpinteria, CA), Wilm’s tumor-1 (WT1) (Santa Cruz #sc-192), Platelet-derived growth factor receptor-*β* (Pdgfr*β*) (eBiosciences, Millipore #14-1402), *α*-smooth muscle actin (SMA)-FITC (Sigma #F3777), Renin (Santa Cruz #sc-27318), Lef1 (Cell Signaling #2230), Pecam (BD Pharmingen #BDB550274, San Jose, CA), Bcl2-like 11 (Bim) (Cell Signaling #2819), Meis1/2/3 (Millipore #05-779), aCaspase3 (Promega #PRG7481, Madison, WI), and phospho-histone H3 (Sigma #H9161). TSA amplification was used for Yap, pYap, and Anxa2 antibodies. Sequential immunofluorescence staining followed by goat anti-Rabbit IgG blocking (Jackson ImmunoResearch #111-007-003) was performed for coimmunofluorescence with multiple antibodies raised in rabbit. All immunofluorescence staining was performed on at least three sets of E18.5 embryos for controls and mutants from three different litters, and images were acquired on a Leica DM2500 microscope equipped with a Qimaging QICAM Fast 1394 camera, and rotated for representation purposes. All image quantification was performed with the use of ImageJ.

### Taqman miRNA arrays

miRNA profiling was performed using Taqman quantitative miRNA assays in a 384-well microfluidic card format (Life Technologies). To isolate FoxD1^+^ cells and their cellular derivatives for profiling, we performed fluorescence-activated cell sorting (FACS) using E15.5 *FoxD1*^*GC*^*;tdTomato;Dicer*^*fl/+*^ kidneys, which permanently express the tdTomato fluorescent protein in all cells derived from the FoxD1^+^ lineage. The tdTomato-positive cells were sorted directly into TRIzol (Life Technologies), and total RNA, including the miRNA fraction, was purified using the Direct-zol RNA MiniPrep Kit (Zymo Research, Irvine, CA). Two sets of pooled total RNA were submitted to the University of Pittsburgh Genomics Research Core for miRNA quantitative PCR profiling using the TaqMan Array Rodent MicroRNA A Card Set v2.0 (Life Technologies) with preamplification. The data were analyzed in R using an HTqPCR package (Bioconductor) (Dvinge and Bertone 2009) and miRNA *C*_T_ values normalized against the U6snRNA. miRNAs enriched in the renal stroma were identified with a raw *C*_T_ value of <30. Locked nucleic acid (Exiqon) section in situ hybridization (LNA-SISH) was performed to validate the miRNA qPCR data, as previously described (Ho et al. 2011).

### Illumina microarray analysis

Whole-transcriptome microarray profiling was performed using an Illumina MouseWG-6 v2.0 chip on three litters of E15.5 and E18.5 kidneys. Each litter consists of kidneys pooled from two embryos per genotype (*FoxD1*^*GC*^*;Dicer*^*fl/fl*^, and Cre-negative littermate controls), resulting in a total of six biological replicates per genotype being assayed for each time point. Total RNA was extracted with the RNeasy Mini Kit (Qiagen, Valencia, CA), followed by a Bioanalyzer test using an Agilent RNA 6000 Nano Kit to assess the integrity of the RNA. All Bioanalyzer quality control, labeling, hybridization, and array scanning were performed at the University of Pittsburgh Genomics Research Core. The microarray data were analyzed as previously described (Phua et al. 2013), using *lumi* and *limma* packages from Bioconductor in R (Smyth 2004; Du et al. 2008). Litter differences were factored into the linear model fit formula, and genes with a *B*-statistics value of >0 are considered statistically significant. Pathway analyses were performed using ToppFun (Chen et al. 2009), Gene Set Enrichment Analysis (Subramanian et al. 2005), and GENE-E (Broad Institute). miRNA:mRNA interactions were analyzed in ingenuity pathway analysis.

Quantitative real-time PCR was performed using standard SYBR Green assay on a BioRad CFX96 Touch Real-Time PCR Detection instrument. Cycle number (*C*_q_) was obtained through regression analysis mode, followed by normalization to *glyceraldehyde 3-phosphate dehydrogenase* (*Gapdh)* as the reference gene. Primers used for the microarray validation were designed to encompass the microarray probe sequence using Primer-BLAST, and are listed in Table S1. The microarray data are available on GEO under the following accession number: GSE64442. Primers used to evaluate the excision of *Dicer* exon 24 are listed in Table S6. Section in situ hybridization validation of the microarray was performed as previously described using riboprobes that were PCR-synthesized from whole-embryo cDNA (Rumballe et al. 2011). Sequencing was performed using T7 primers, followed by nucleotide-BLAST to confirm gene specificity. Primers used for the riboprobe synthesis were designed to encompass the microarray probe using Primer-BLAST (Ye et al. 2012), and are listed in Table S2.

### Western blotting

Total protein was extracted from three litters of E18.5 kidneys using radioimmunoprecipitation assay (RIPA) buffer supplemented with cOmplete protease inhibitor cocktail (Roche, Indianapolis, IN). Protein lysates were separated on an Any kD™ Precast SDS-PAGE gel (BioRad, Hercules, CA), transferred to a PVDF membrane, blocked with 5% nonfat milk and probed with the following antibodies: Bcl2-like 11 (Bim) (Cell Signaling #2819), p53 (Santa Cruz #sc-6243), and Gapdh (Trevigen #2275-PC-100). Detection was performed using ECL Plus Substrate (Thermo Scientific, Waltham, MA), followed by exposure to X-ray films. Antibodies were stripped from the membrane using Restore Western Blot Stripping Buffer (Thermo Scientific) prior to subsequent immunodetections.

## Results

### Loss of stromal miRNAs results in a multifaceted renal phenotype

**Figure 1:**
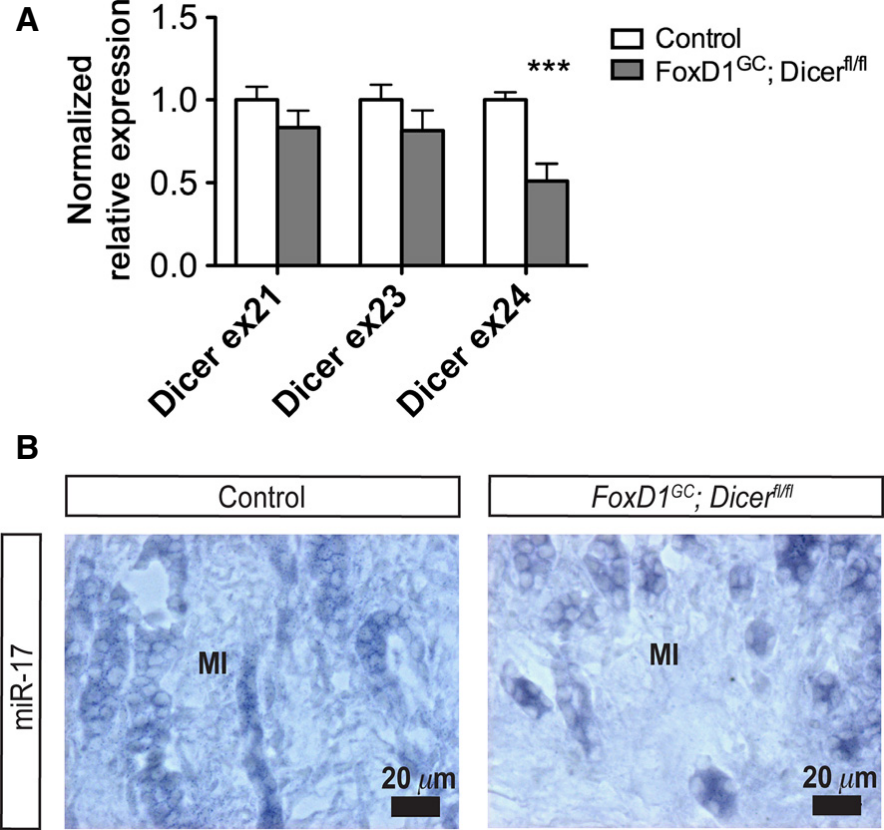
*Dicer* exon 24 excision resulted in impaired miRNA biogenesis in E18.5 *FoxD1*^*GC*^*;Dicer*^*fl/fl*^ kidneys. (A) To evaluate the excision of *Dicer* exon 24, quantitative real-time PCR was carried out and revealed a significant decrease in *Dicer* exon 24, but not in exons 21 and 23 transcripts (*n *=* *6, ***P <* *0.001). (B) LNA SISH revealed reduced miR-17 expression within the *FoxD1*^*GC*^*;Dicer*^*fl/fl*^ medullary interstitium (MI), thereby indicating impaired miRNA biogenesis as a consequence of loss of Dicer activity.

The *FoxD1*-positive cortical renal stroma in the developing kidney marks a stromal progenitor population that gives rise to diverse cell lineages, including the cortical and medullary interstitial cells, glomerular mesangial cells, and pericytes (Kobayashi et al. 2014). To delineate the role of stromal miRNAs in renal development, we ablated *Dicer*, an endoribonuclease required for miRNA biogenesis, in the renal stroma and its derivatives by generating *FoxD1*^*GC*^*;Dicer*^*fl/fl*^ transgenic mice (Harfe et al. 2005; Humphreys et al. 2010). To evaluate the excision of *Dicer* exon 24 from the *Dicer*^*fl*^ allele by the *FoxD1*^*GC*^ allele, we performed quantitative real-time PCR and observed a significant decrease in exon 24 (***P <* *0.001) but not exons 21 and 23 transcripts in our mutant model (Fig.1A). Moreover, LNA SISH showed reduced miR-17 expression within the FoxD1^+^ derived medullary interstitium, indicative of impaired miRNA biogenesis in our model (Fig.1B).

**Figure 2:**
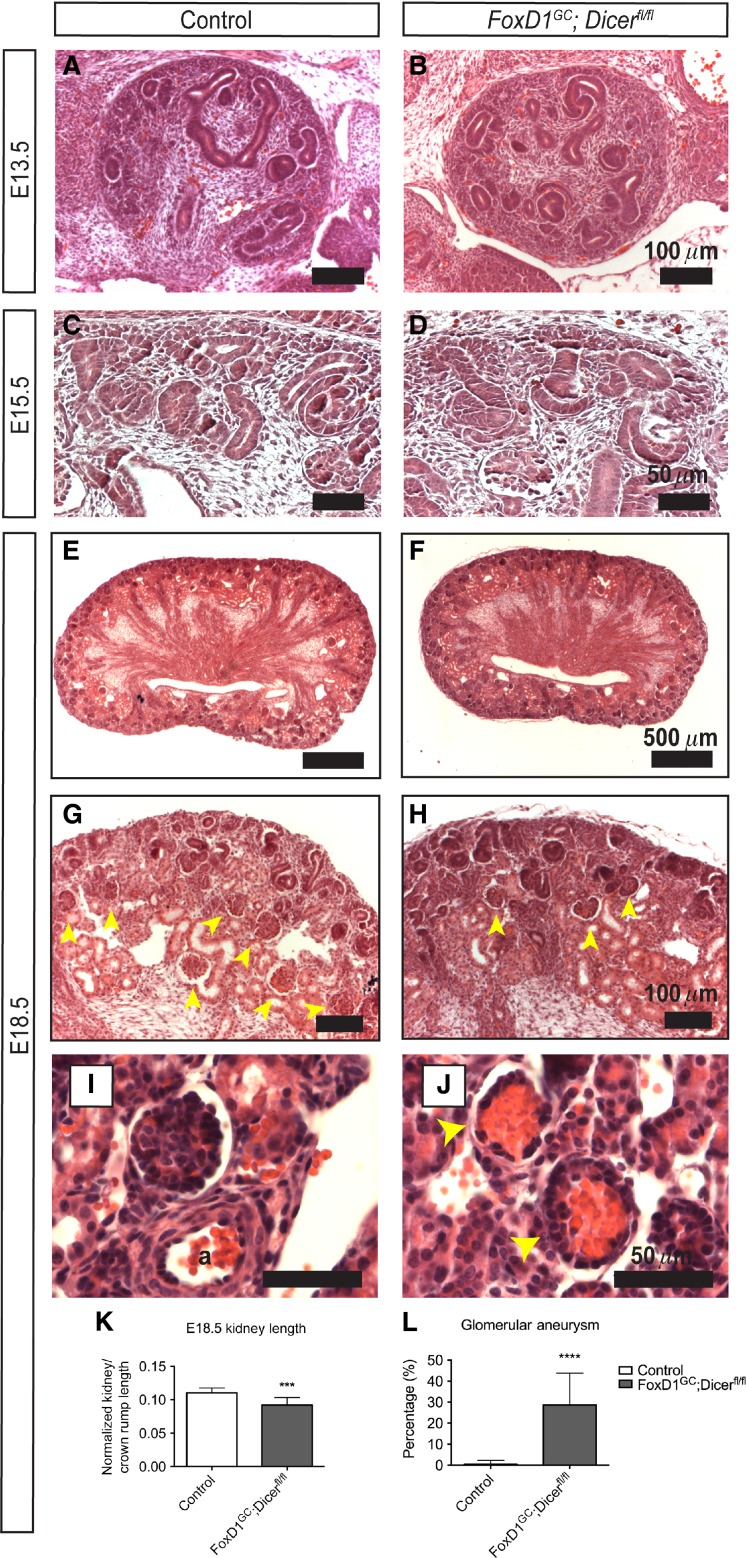
Loss of stromal miRNAs resulted in renal hypodysplasia in *FoxD1*^*GC*^*;Dicer*^*fl/fl*^ kidneys. (A, B) Histological analysis of E13.5 kidneys revealed no overt defects in *FoxD1*^*GC*^*;Dicer*^*fl/fl*^ kidneys. (C, D) Similarly, no obvious defects were noted histologically in E15.5 control and *FoxD1*^*GC*^*;Dicer*^*fl/fl*^ kidneys, with the appearance of developing nephrons. (E, F) At E18.5, a patent renal papilla was observed in both control and *FoxD1*^*GC*^*;Dicer*^*fl/fl*^ kidneys. (G, H) In comparison to controls, *FoxD1*^*GC*^*;Dicer*^*fl/fl*^ kidneys exhibited fewer developing glomeruli (yellow arrowheads) and a disorganized nephrogenic zone at E18.5. (I, J) A subset of juxtamedullary *FoxD1*^*GC*^*;Dicer*^*fl/fl*^ glomeruli demonstrated an microaneurysmal phenotype at E18.5 (yellow arrowheads). a, artery. (K) At E18.5, *FoxD1*^*GC*^*;Dicer*^*fl/fl*^ kidneys were smaller than control counterparts, as measured by renal length normalized to crown–rump length. ***P <* *0.001 (Student’s *t*-test). (L) Since glomerular microaneurysms occur only within the juxtamedullary region, semiquantitative counting of 352 control and 406 *FoxD1*^*GC*^*;Dicer*^*fl/fl*^ juxtaglomerular glomeruli (*n *=* *3 kidneys for both controls and mutants) showed that approximately 28 ± 15% of *FoxD1*^*GC*^*;Dicer*^*fl/fl*^ glomeruli had microaneurysms as denoted by atypical capillary ballooning with loss of patent capillary loops. ****P <* *0.0001. Histological analysis was performed on at least three control and mutant embryos.

Previous studies have shown that the *FoxD1*^*GC*^ allele is expressed as early as embryonic day 10.5 (E10.5) (Kobayashi et al. 2014). However, histological analysis of embryonic day E13.5 and E15.5 *FoxD1*^*GC*^*;Dicer*^*fl/fl*^ kidneys revealed no overt disruption to the initial events of renal development, with the appearance of condensing nephron progenitors around ureteric bud tips, and the formation of early developing nephron structures, such as renal vesicles (Fig.2A–D). By E18.5, *FoxD1*^*GC*^*;Dicer*^*fl/fl*^ embryos developed renal hypodysplasia, with fewer developing nephron structures, in the context of a disorganized nephrogenic zone (Fig.2E–H). The renal length/crown–rump ratio was significantly decreased in mutant embryos, suggesting that the renal hypodysplasia is more likely to reflect a direct requirement for Dicer activity in the renal stroma during nephrogenesis rather than global developmental delay (Fig.2K).

Interestingly, histological analysis of *FoxD1*^*GC*^*;Dicer*^*fl/fl*^ kidneys also demonstrated glomerular capillary microaneurysms, most often in mature juxtamedullary glomeruli (Fig.2I and J). Semiquantitative counting of glomeruli from control (*n *=* *3) and *FoxD1*^*GC*^*;Dicer*^*fl/fl*^ (*n *=* *3) midsagittal sections showed that approximately 29 ± 15% of *FoxD1*^*GC*^*;Dicer*^*fl/fl*^ glomeruli contained microaneurysms (****P <* *0.0001) (Fig.2L). This observation suggests that miRNAs in the glomerular mesangium play a role in glomerular vascular maintenance upon maturation, and that the onset of the defect is a progressive event that occurs during late-stage nephrogenesis. Together, these results suggest that stromal miRNAs are likely to have important roles in regulating the renal cortical interstitium, which comprises the nephron progenitor niche, and vascular supporting cells derived from FoxD1-positive cells, such as the glomerular mesangium.

### Lack of stromal miRNAs affects both the stromal and nephron progenitor compartments in kidney development

**Figure 3:**
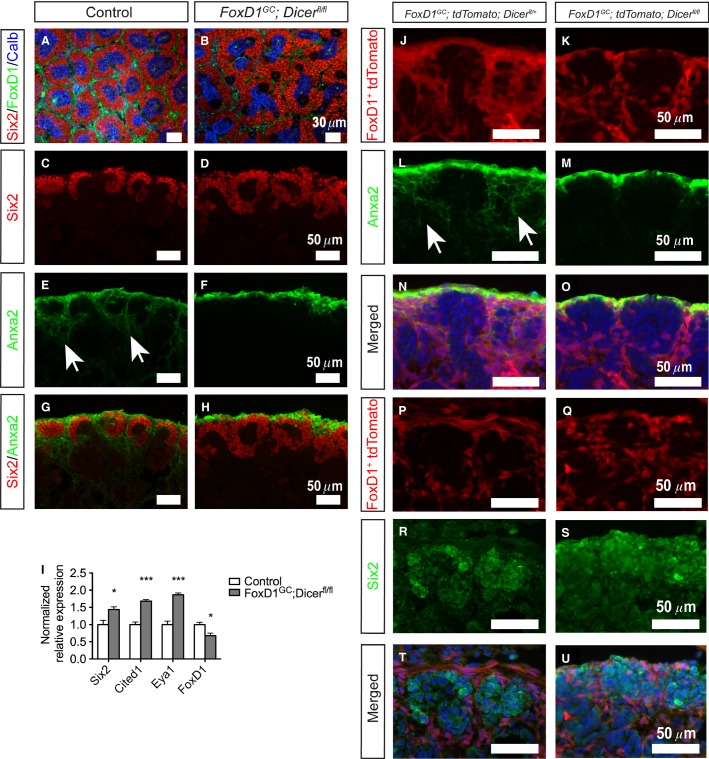
Loss of stromal miRNAs resulted in expansion of nephron progenitors and an intrinsic stromal defect. (A, B) Whole-mount immunofluorescence staining for Six2 (nephron progenitor), FoxD1 (renal stroma), and Calbindin (ureteric tips) revealed disorganization and expansion of nephron progenitors surrounding the ureteric tips, with decreased FoxD1^+^ cells, in E18.5 *FoxD1*^*GC*^*;Dicer*^*fl/fl*^ kidneys. (C–H) Section immunofluorescence also reaffirmed the aberrant expansion of Six2^+^ (red) nephron progenitors, and loss of Anxa2^+^ (green) stroma in the interdigitating renal cortical stroma of E18.5 *FoxD1*^*GC*^*;Dicer*^*fl/fl*^ kidneys (white arrows). (I) Quantitative real-time PCR performed on control and *FoxD1*^*GC*^*;Dicer*^*fl/fl*^ E18.5 kidneys confirmed increased levels of *Six2, Cited1,* and *Eya1* expression in nephron progenitor and reduction of *FoxD1* transcripts. *n *=* *6, *P <* *0.05, ***P <* *0.001. (J–O) Lineage tracing of the renal stroma performed with *tdTomato* reporter mice (red) demonstrated that while lineage tagged *FoxD1*^*GC*^*;Dicer*^*fl/fl*^ stroma (*FoxD1*^*GC*^*;tdTomato;Dicer*^*fl/fl*^*)* adopts the expression pattern observed in E18.5 *FoxD1*^*GC*^*;tdTomato;Dicer*^*fl/+*^ kidneys (white arrows), the interdigitating stroma lacked Anxa2 expression (green). (P–U) Lineage tracing and Six2 immunostaining at E18.5 revealed that expansion of *FoxD1*^*GC*^*;Dicer*^*fl/fl*^ Six2^+^ nephron progenitors is not due to transdifferentiation of the FoxD1^+^ renal stroma into nephron progenitors. Immunofluorescent studies were performed on at least three control and mutant embryos.

The renal stroma functions as a critical microenvironment for nephron progenitors, and is the source of signals that regulate the self-renewal and differentiation of nephron progenitors (Das et al. 2013). To address the effects of loss of Dicer activity in the renal stroma on the interaction between the FoxD1^+^ stromal cells adjacent to nephron progenitors, we first performed whole-mount immunofluorescence staining for Six2 (nephron progenitor) (Kobayashi et al. 2008), FoxD1 (renal stroma) (Kobayashi et al. 2014), and Calbindin (ureteric bud) from E13.5 to E18.5. Subtle increases in Six2^+^ nephron progenitors at E15.5 were noted, and by E18.5, the *FoxD1*^*GC*^*;Dicer*^*fl/fl*^ kidneys displayed a clear expansion, and disorganization, of Six2^+^ nephron progenitors around ureteric tips and reduced FoxD1^+^ stroma, in comparison to the well-defined nephron progenitor niches composed of FoxD1^+^ renal stroma in control kidneys (Fig.3A and B). Ureteric branching morphogenesis also appeared to be perturbed in the absence of stromal miRNAs (Fig.3A and B). In accord with the whole-mount staining, quantitative real-time PCR verified the increase in *Six2* (*P <* *0.05) and decrease in *FoxD1* transcripts (*P <* *0.05) (Fig.3I). Two other markers of nephron progenitors, *Cited1* (***P <* *0.001) and *Eya1* (***P <* *0.001), also demonstrated increased transcript levels by quantitative real-time PCR, suggesting that there is an overall increase in the number of nephron progenitors, rather than simply increased Six2 expression in progenitors (Fig.3I) (Kalatzis et al. 1998; Boyle et al. 2008).

**Figure 4:**
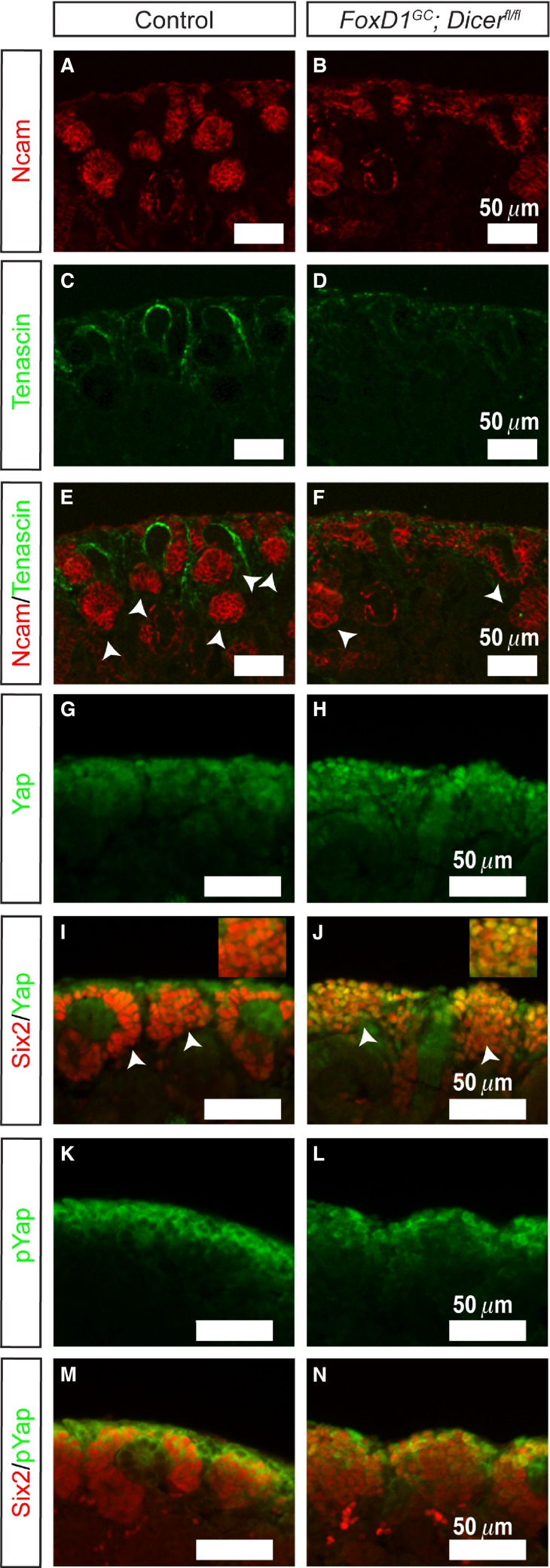
*FoxD1*^*GC*^*;Dicer*^*fl/fl*^ kidneys demonstrate fewer developing nephrons, and dysregulation of Yap signaling in *FoxD1*^*GC*^*;Dicer*^*fl/fl*^ nephron progenitors. (A–F) E18.5 *FoxD1*^*GC*^*;Dicer*^*fl/fl*^ kidneys exhibited fewer Ncam^+^ differentiating nephrons (arrowheads) (red), amid loss of interdigitating Tenascin^+^ (green) renal cortical stroma. (G–J) Immunofluorescence staining for Yap (green) showed increased nuclear Yap localization in the E18.5 *FoxD1*^*GC*^*;Dicer*^*fl/fl*^ Six2^+^ nephron progenitors (red). The insert illustrates a higher magnification of the image. (K–N) pYap staining (green), however, revealed no differences between the E18.5 *FoxD1*^*GC*^*;Dicer*^*fl/fl*^ and control kidneys. Immunostaining was performed on at least three control and mutant embryos.

To further define the effects of loss of Dicer activity in the renal cortical stroma, section immunofluorescence staining was performed for Annexin2 (Anxa2), a newly identified renal stromal marker (Brunskill et al. 2014). Although control kidneys showed well-defined Six2^+^ nephron progenitor populations with interdigitating Anxa2^+^ stroma, *FoxD1*^*GC*^*;Dicer*^*fl/fl*^ kidneys showed expansion of Six2^+^ cells accompanied by a lack of interdigitating Anxa2^+^ stroma (Fig.3C–H). Of note, expression of Anxa2 in the renal capsule appeared to be unaffected in the *FoxD1*^*GC*^*;Dicer*^*fl/fl*^ kidneys (Fig.3E–H). In accord with the Anxa2^+^ staining, the expression of another renal stromal marker, Tenascin, demonstrated decreased and disorganized expression between nephron progenitors (Fig.4A–F). Lineage tracing was performed by generating *FoxD1*^*GC*^*;tdTomato;Dicer*^*fl/fl*^ mice, which permanently express tdTomato in all cells derived from the FoxD1^+^ lineage. Fate mapping of the FoxD1^+^ lineage in *FoxD1*^*GC*^*;Dicer*^*fl/fl*^ kidneys showed that while tdTomato^+^ cells were appropriately localized in the nephrogenic zone with no evidence for overt transdifferentiation into nephron progenitors, the interdigitating tdTomato^+^ cells between nephron progenitors lacked Anxa2 expression (Fig.3J–U). This finding suggests that there exists an Anxa2^+^ subpopulation in the renal cortical stroma that is differentially affected by loss of miRNAs in FoxD1-derived cells. The functional significance of this population remains unknown.

Aside from the stromal defects, *FoxD1*^*GC*^*;Dicer*^*fl/fl*^ kidneys demonstrate an increase in Six2^+^ cells, indicating a noncell autonomous effect on nephron progenitors. This observation is reminiscent of the *stromaless* mice phenotype, whereby ablation of the renal stroma resulted in an expansion of the nephron progenitor population (Das et al. 2013; Hum et al. 2014). The aberrant expansion of Six2^+^ nephron progenitors in *FoxD1*^*GC*^*;Dicer*^*fl/fl*^ kidneys could arise from an impaired transition of the Six2^+^ cells into epithelialized nephrons. While Ncam staining showed evidence for developing nephrons, the numbers of Ncam^+^ structures were fewer than their control counterparts, so we cannot exclude at least a partial defect in differentiation (Fig.4A–F) (Klein et al. 1988). Similar to the *stromaless* mice, the *FoxD1*^*GC*^*;Dicer*^*fl/fl*^ kidneys displayed increased nuclear Yap accumulation in Six2^+^ cells (Fig.4G–J). In contrast, pYap localization within the *FoxD1*^*GC*^*;Dicer*^*fl/fl*^ Six2^+^ cells appears unaffected (Fig.4K–N), indicating that the nephron progenitors were still responsive toward stroma-Fat4/Yap signaling in the absence of miRNAs, and therefore, potentially retain the competency to undergo differentiation into epithelialized structures.

### Glomerular mesangial defects in *FoxD1*^*GC*^*;Dicer*^*fl/fl*^ kidneys

**Figure 5:**
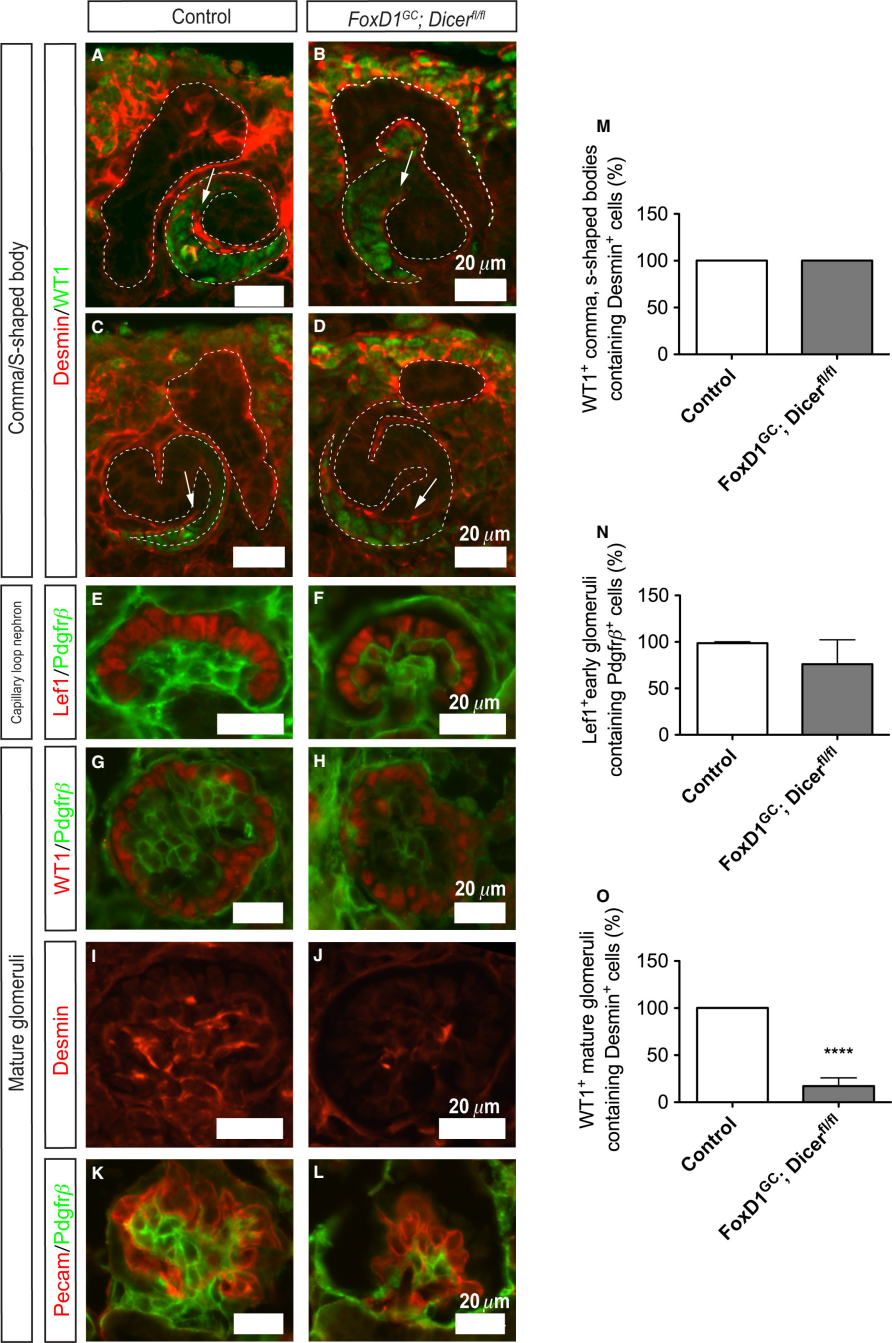
Mesangial defects in *FoxD1*^*GC*^*;Dicer*^*fl/fl*^ mature glomeruli at E18.5. (A–D) Immunofluorescence staining for Desmin (red) showed evidence for early mesangial cell migration in both control (*n *=* *3 embryos, 25 comma-/s-shaped bodies) and *FoxD1*^*GC*^*;Dicer*^*fl/fl*^ (*n *=* *3 embryos, 16 comma-/s-shaped bodies) developing WT1^+^ (red) comma- and s-shaped bodies. (E, F) Developing capillary loop nephrons as marked by expression of Lef1 (red) in early podocytes showed evidence for Pdgfr*β*^+^ (green) mesangial formation in *FoxD1*^*GC*^*;Dicer*^*fl/fl*^ kidneys (*n *=* *3 embryos, 49 glomeruli), although the mesangial network appeared simplified when compared to controls (*n *=* *3 embryos, 52 glomeruli). (G–L) Mature *FoxD1*^*GC*^*;Dicer*^*fl/fl*^ glomeruli displayed overt mesangial abnormalities as evident by a significant reduction in Pdgfr*β*^+^ (green) and Desmin^+^ (red) immunoreactivity. (M–O) Semiquantitative analysis revealed no significant difference in developing WT1^+^ comma- and s-shaped bodies (M) or Lef1^+^ early glomeruli (N) in control and *FoxD1*^*GC*^*;Dicer*^*fl/fl*^ embryos. However, there were significantly fewer WT1^+^ mature glomeruli with a Desmin^+^ mesangial network in *FoxD1*^*GC*^*;Dicer*^*fl/fl*^ kidneys (*n *=* *3 embryos, 29 glomeruli, ****P <* *0.0001), when compared to controls (*n *=* *3 embryos, 19 glomeruli) (O). The presence of WT1^+^ (red) podocytes and intussuscepted Pecam^+^ (red) capillaries in the *FoxD1*^*GC*^*;Dicer*^*fl/fl*^ glomeruli suggest that the mesangial defects are likely to be a primary consequence of loss of stromal miRNAs.

Glomerular mesangial cells are specialized pericytes derived from the renal stroma that function as a dynamic support structure for glomerular endothelial cells (Lindahl et al. 1998). Formation of the glomerular vasculature begins when both endothelial and mesangial cells are recruited into the developing comma-shaped bodies, progressing into s-shaped bodies, and eventually capillary loop nephron as development proceeds (Lindahl et al. 1998). Notch signaling is indispensable for the initial migration of presumptive mesangial cells into the developing comma- and s-shaped bodies (Boyle et al. 2014; Lin et al. 2014). To address whether the mesangial defects in *FoxD1*^*GC*^*;Dicer*^*fl/fl*^ glomeruli were due to a failure in migration of mesangial cells, immunostaining was performed, and demonstrated the presence of Desmin^+^ mesangial cells migrating into the developing comma- and s-shaped bodies, with the proximal segments marked by WT1 staining (Fig.5A–D). This was also followed by successive progression into capillary loop nephrons, whereby glomerular capillary intussusception was accompanied by formation of the Pdgfr*β*^+^ mesangium network within Lef1^+^ early podocytes. Notably, there was decreased capillary looping in *FoxD1*^*GC*^*;Dicer*^*fl/fl*^ glomeruli as compared to the controls (Fig.5E and F). Interestingly, *FoxD1*^*GC*^*;Dicer*^*fl/fl*^ mature glomeruli exhibited an intrinsic mesangial defect, accompanied by a reduction in the mesangial markers, Pdgfr*β* and Desmin (Fig.5G–L). The mesangial defect, however, appears to be an intrinsic abnormality as a consequence of a loss of stromal miRNAs, since WT1^+^ podocytes and Pecam^+^ endothelium continued to be present in defective glomeruli (Fig.5G–J). Collectively, the results demonstrated that the *FoxD1*^*GC*^*;Dicer*^*fl/fl*^ mesangial abnormality was not due to a defect in initial specification of mesangial cells or vascular migration during nephrogenesis, but rather an inability to promote cellular survival.

### Stromal miRNAs are required for the establishment and maintenance of multiple renal vascular-related compartments

**Figure 6:**
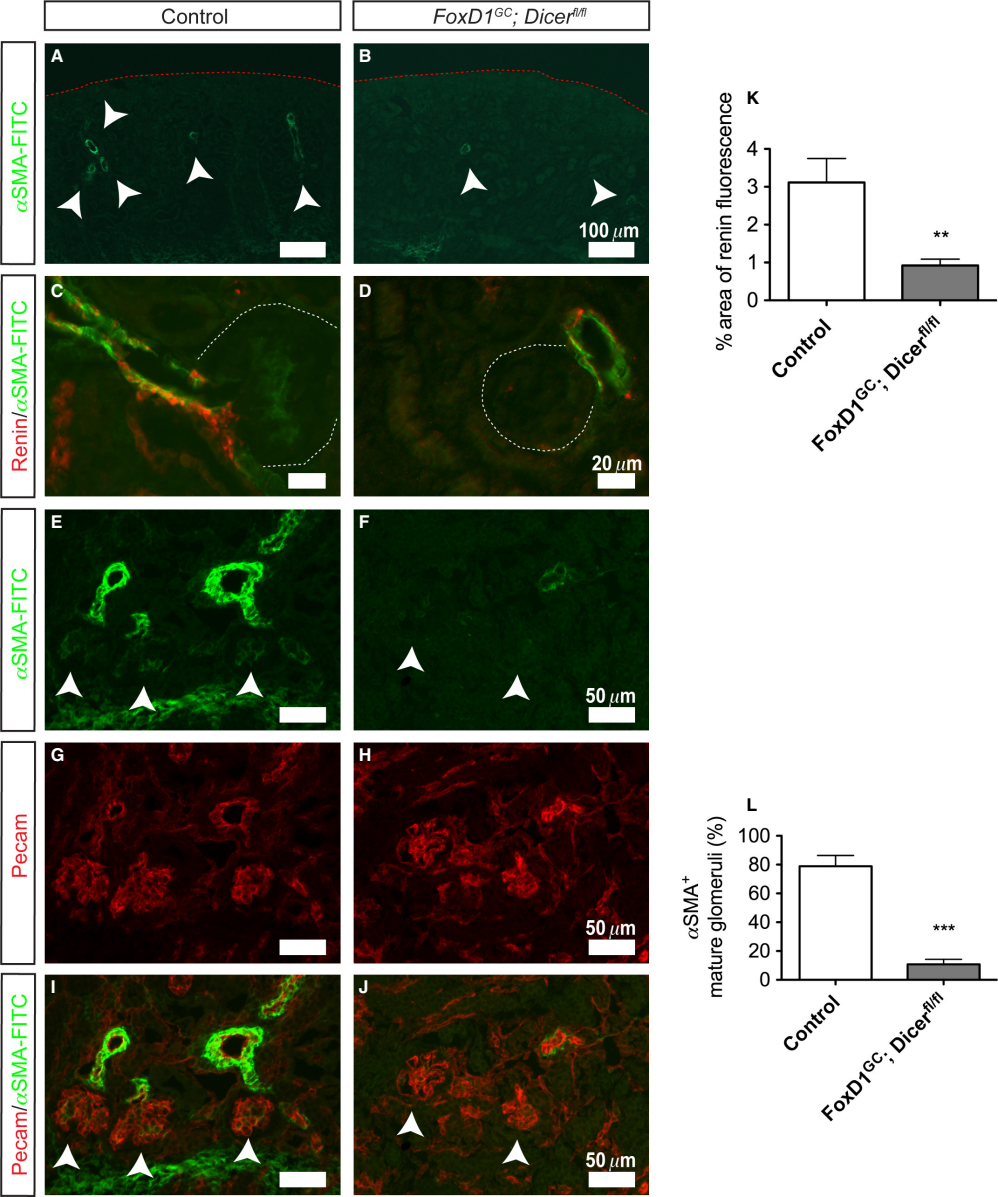
Reduced smooth muscle arterioles and renin-expressing cells in *FoxD1*^*GC*^*;Dicer*^*fl/fl*^ kidneys. (A, B) Immunofluorescence staining for *α*SMA-FITC conjugate showed a significant reduction of smooth muscle arteries in E18.5 *FoxD1*^*GC*^*;Dicer*^*fl/fl*^ kidneys (arrowheads). (C,D, and K) Remnant *FoxD1*^*GC*^*;Dicer*^*fl/fl*^ glomerular smooth muscle arterioles at E18.5 exhibited a significant reduction in renin-expressing cells (red) (*n *=* *3 embryos, 15 control and 16 *FoxD1*^*GC*^*;Dicer*^*fl/fl*^ optical images at 40× magnification, **P <* *0.01). (E–J and L) Immunofluorescence staining for the *α*SMA-FITC conjugate demonstrates a reduction in expression associated with glomeruli. Consistent with a mesangial defect, *FoxD1*^*GC*^*;Dicer*^*fl/fl*^ glomeruli lacked expression of *α*SMA-FITC^low^ as compared to controls (arrowheads) (*n *=* *3 embryos, 43 control and 73 *FoxD1*^*GC*^*;Dicer*^*fl/fl*^ mature glomeruli, ***P <* *0.001). Staining for Pecam (red) was used to identify blood vessels and glomerular endothelial cells.

Besides formation of the glomerular mesangial network, FoxD1^+^ stroma gives rise to multiple vascular supportive cell types including capillary pericytes and components of the juxtaglomerular apparatus (afferent arteriole smooth muscle and renin-expressing cells) (Kobayashi et al. 2014). Even accounting for the reduced number of glomeruli in *FoxD1*^*GC*^*;Dicer*^*fl/fl*^ kidneys, both *α*SMA and Ren1 immunostaining revealed a decrease in afferent arteriole smooth muscle and renin-expressing cells at the glomerular vascular pole of *FoxD1*^*GC*^*;Dicer*^*fl/fl*^ kidneys as compared to the controls (Fig.6A–D). Additionally, *FoxD1*^*GC*^*;Dicer*^*fl/fl*^ glomeruli lacked *α*SMA^low^ expression in the mesangium, providing further evidence for a mesangial defect (Fig.6E–J).

### miRNA expression profile in the renal stroma and derivatives

**Figure 7:**
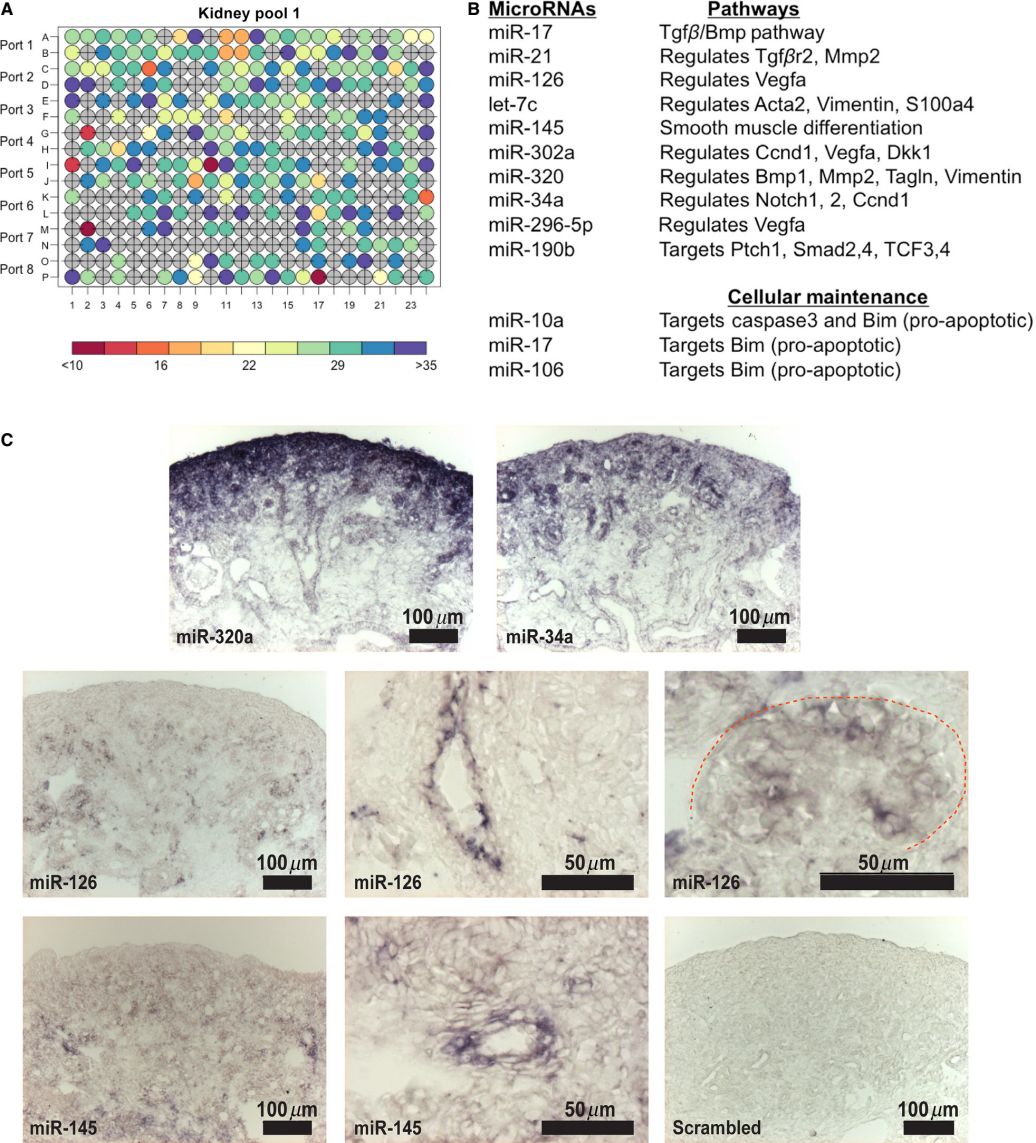
miRNA expression profile of the renal stroma. (A) High-throughput miRNA TaqMan quantitative PCR profiling of two sets of pooled renal stroma revealed enrichment of 125 of 375 miRNAs assayed based on a *C*_T_ value of <30 at E15.5. (B) A subset of miRNAs is associated with key regulatory pathways involved in vascular development and cellular development. (C) LNA-SISH showed expression of miRs-320a, 34a, 126, and 145 in renal stroma and/or their cellular derivatives in E18.5 wild-type kidneys. miRs-320a and 34a were also present in nephron progenitors, ureteric bud, and epithelial tubules. On the other hand, miRs-126 and 145 displayed a more restrictive expression pattern, confined to the smooth muscle arteries, glomerular vasculature, and blood vessels (for miR-126).

The results thus far have demonstrated that loss of stromal miRNAs results in a multifaceted renal phenotype with defects in the nephron progenitor niche, glomerular mesangium, smooth muscle arterioles, and renin-expressing cells. To identify candidate miRNAs that could be modulating this phenotype, we performed FACS for tdTomato^+^ labeled cells from E15.5 *FoxD1*^*GC*^*;Dicer*^*fl/+*^*;tdTomato* kidneys for miRNA qPCR profiling, the earliest time point at which a phenotype was noted (Fig.2). Of the 375 miRNAs assayed by TaqMan miRNA Array, 125 showed enrichment in the renal stroma and its derivatives based on *C*_T_ cutoff value of <30 (Fig.7A) (Table S3). These miRNAs included several miRNAs involved in vascular development (miRs-34a, 126, 145, 296-5p 302a, and 320) (Jeyaseelan et al. 2008; Cordes et al. 2009; Larsson et al. 2009; Liu et al. 2009; Pang et al. 2010; Vaira et al. 2012), members of the *miR-17˜92* cluster (miRs-17, 19a, 19b, 20a, 20b, 92a), and apoptosis-related miRNAs (miRs-10a, 17 and 106) (Ho et al. 2011) (Fig.7B). To validate the miRNA profiling data, we performed locked nucleic acid section in situ hybridization (LNA-SISH) and corroborated the expression for miRs-320a, 34a, 126, and 145 (Fig.7C). Besides the stromal expression for miRs-320a and 34a, these miRNAs were also present in nephron progenitors, ureteric bud, and epithelial tubules (Fig.7C). On the other hand, miRs-126 and 145 displayed a more restrictive expression pattern, confined to the smooth muscle arteries, glomerular vasculature, and blood vessels (for miR-126) (Fig.7C).

### Stromal miRNAs are required for maintenance of its cellular descendants

To comprehensively define the transcriptional changes that may be modulating the defects in the *FoxD1*^*GC*^*;Dicer*^*fl/fl*^ kidneys, we performed whole-transcriptome microarray profiling on E15.5 and E18.5 *FoxD1*^*GC*^*;Dicer*^*fl/fl*^ and control kidneys. A total of 717 genes were identified to be differentially expressed in E18.5 *FoxD1*^*GC*^*;Dicer*^*fl/fl*^ kidneys based on a on *B*-statistics > 0 (Table S4). In contrast, profiling of E15.5 *FoxD1*^*GC*^*;Dicer*^*fl/fl*^ kidneys revealed 116 differentially expressed genes (Table S5).

Using GUDMAP gene expression datasets (Harding et al. 2011), we further subdivided the E18.5 microarray data into different developmental renal cell types based on gene expression information in the different renal compartments curated by GUDMAP. In this manner, 36 stroma-, 71 renin-, 49 mesangium-, and 17 nephron progenitor-associated genes were identified. Several of these differentially expressed genes, including elevated nephron progenitor markers (*Six2*, *Cited1,* and *Eya1*) and reduced stroma-associated genes (*Ren1, Acta2, Meis1, Notch3*, and *Pdgfrα*), were in agreement with our earlier observations, thereby validating the microarray data.

### Loss of stromal miRNAs resulted in dysregulation of Bim and p53 effector genes, leading to increased apoptosis

**Figure 8:**
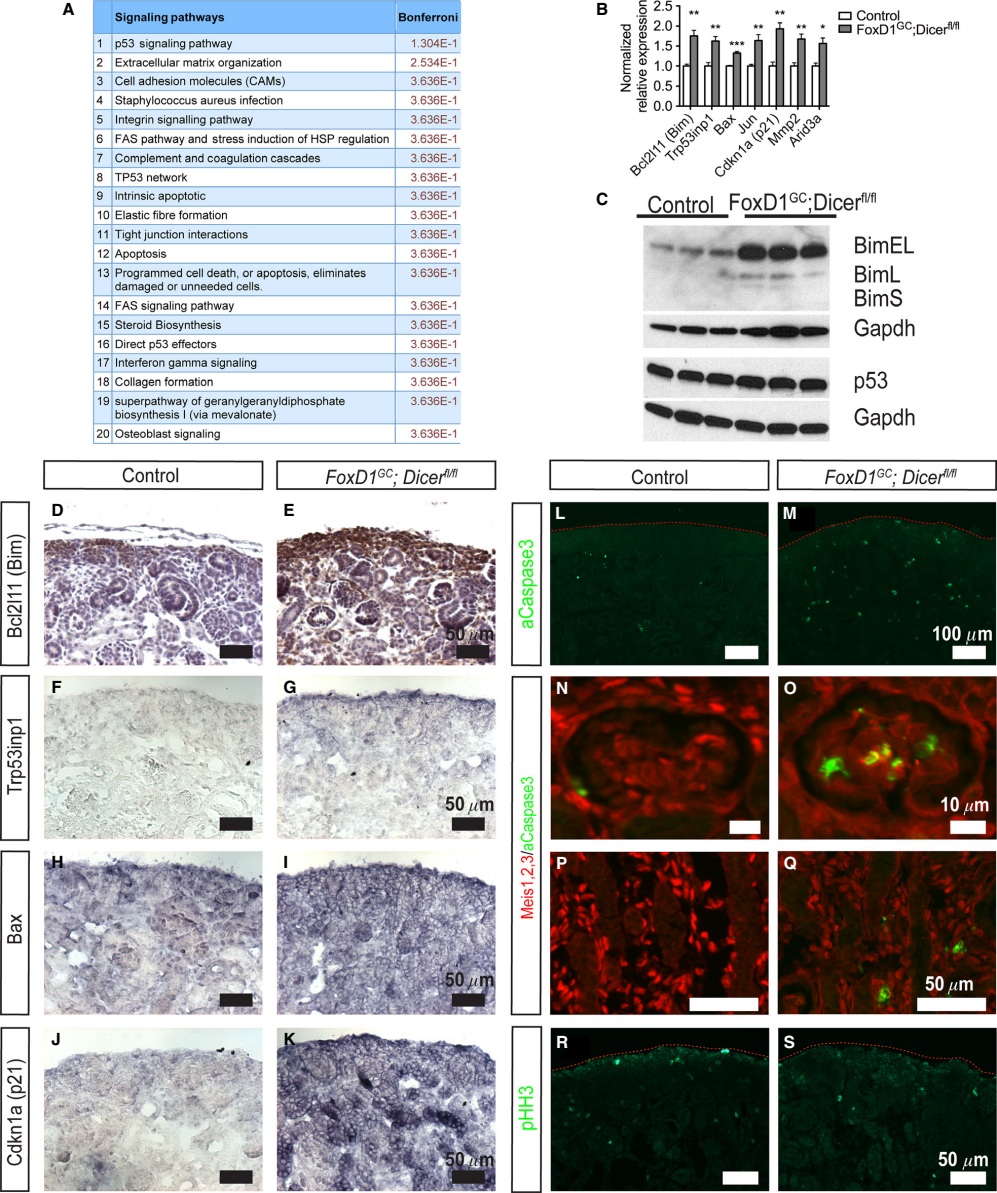
Increased apoptosis in *FoxD1*^*GC*^*; Dicer*^*fl/fl*^ stromal derived cells. (A) ToppFun analysis of differentially expressed transcripts obtained from E18.5 control and *FoxD1*^*GC*^*; Dicer*^*fl/fl*^ kidneys by microarray showed evidence of increased expression of transcripts associated with apoptosis and dysregulation of the p53 signaling pathway. (B) Quantitative PCR validated the upregulation of *Bcl2 l11 (Bim)* and genes associated with p53 signaling including *Trp53inp1, Bax, Jun, Cdkn1a (p21), Mmp2,* and *Arid3a* in E18.5 *FoxD1*^*GC*^*; Dicer*^*fl/fl*^ kidneys. *n *=* *6, *P <* *0.05, **P <* *0.01, ***P <* *0.001. (C) Western blot analysis showed increased levels of BimEL and BimL but normal levels of p53 in E18.5 *FoxD1*^*GC*^*; Dicer*^*fl/fl*^ kidneys. (D, E) Section immunohistochemistry for confirmed increased and ectopic Bim expression (brown) in the renal stroma and nephron progenitors. (F–K) Section in situ hybridization revealed increased and ubiquitous expression of *Trp53inp1, Bax,* and *p21* in E18.5 *FoxD1*^*GC*^*; Dicer*^*fl/fl*^ kidneys. (L, M) Immunofluorescence revealed increased aCaspase3^+^ (green) apoptotic cells in the E18.5 *FoxD1*^*GC*^*; Dicer*^*fl/fl*^ kidneys. (N–Q) Apoptotic cells were largely localized to Meis1,2,3^+^ (red) glomerular mesangium and interstitial cells. (R, S) No differences in proliferation based on phosphohistone H3 staining were observed. Immunostaining was performed on at least three control and mutant embryos.

By applying Gene Set Enrichment Analysis (GSEA) on the E18.5 microarray data, differentially expressed genes were found to be enriched for those involved in apoptosis (ES: 0.572, Nominal *P*-value: 0). In general agreement with our previous loss of miRNA function studies (Ho et al. 2008, 2011; Chu et al. 2014), absence of stromal miRNAs resulted in increased *Bcl2l11 (Bim)* expression, most notable for the BimEL and BimL proapoptotic protein isoforms, in *FoxD1*^*GC*^*;Dicer*^*fl/fl*^ kidneys (Fig.8B and C) (Table S6). Similarly, immunostaining demonstrated increased Bim protein localized in nephron progenitors, as well as ectopically in adjacent interstitial cells (Fig.8D and E).

ToppFun bioinformatics analysis of the E18.5 microarray data showed that in addition to curating multiple processes that are associated with apoptosis, dysregulation of p53 signaling was predicted to be the top pathway modulating the *FoxD1*^*GC*^*;Dicer*^*fl/fl*^ renal phenotype (Fig.8A). Interestingly, Western blot analysis showed no significant changes in p53 protein in *FoxD1*^*GC*^*;Dicer*^*fl/fl*^ kidneys (Fig.8C). Quantitative real-time PCR on the other hand showed significant upregulation of downstream p53 target genes and apoptosis inducers including, *Bax*, *Trp53inp1*, *Jun*, *Cdkn1a*, *Mmp2*, and *Arid3a* (Fig.8B). Upregulation of these p53 effector genes were also validated through SISH, with *Bax, Trp53inp1,* and *Cdkn1a* exhibiting a ubiquitous expression pattern in the *FoxD1*^*GC*^*;Dicer*^*fl/fl*^ kidneys (Fig.8F–K). Together, the results suggest that loss of stromal miRNAs alleviates repression of p53-associated target genes, rather than modulating p53 protein expression. Interestingly, the stromal miRNA profiling identified a total of 72 miRNA:mRNA target interactions involving 31 miRNAs that were predicted to target these p53-dysregulated genes based on Ingenuity Pathways Analysis and TargetScan. Of these interactions, miRs-15b, 21, 30c, 106a, 125b-5p, 145, 214, 222, 296-5p, members of miR-17∼92 cluster (miRs-18a, 92a), and 302a have previously been shown to target *Arid3a, Cdkn1a, Bax, Jun, Bbc3, Casp6, Mdm2,* and *Apaf1* experimentally based on curation by Ingenuity Pathways (Table S7).

Dysregulation of Bim and p53 effector genes are likely to result in increased apoptosis. Consistent with this, immunostaining for aCaspase3 showed ectopic apoptosis in the *FoxD1*^*GC*^*;Dicer*^*fl/fl*^ mesangium and interstitium (Fig.8L–Q). In contrast, pHH3 (which marks the cell cycle mitosis phase) revealed no significant differences in proliferation, although subtle changes in the cell cycle cannot be excluded (Fig.8R and S). Taken together, the results suggest that loss of stromal miRNAs results in ectopic apoptosis in the glomerular mesangium and renal interstitium, likely through both increased Bim and p53 target gene expression.

## Discussion

This study provides evidence that miRNA-mediated posttranscriptional regulation in the FoxD1^+^ renal stroma lineage is indispensable for normal nephrogenesis, development of renal stromal derivatives, and maintenance of the glomerular mesangium. The *FoxD1*^*GC*^*; Dicer*^*fl/fl*^ mice develop multifaceted renal anomalies, including reduced nephron endowment, increased nephron progenitors, reduced smooth muscle in afferent arterioles, decreased renin-expressing cells, and progressive mesangial loss, eventually leading to glomerular microaneurysms as characterized by capillary ballooning. In mammals, Dicer is thought to be required predominantly for the processing of mature miRNAs, although it remains possible that a portion of these phenotypes are due to loss of other Dicer-dependent small RNAs (Calabrese et al. 2007; Zhdanova et al. 2011). The requirement for Dicer activity in the renal stroma in normal nephrogenesis and glomerulogenesis is consistent with the recent report of Nakagawa et al. (2015), specifically given the observation of renal hypodysplasia, a reduction in glomerular number and abnormal glomerular development in this model. This study extends those findings to describe the effect of Dicer deletion on nephron progenitors and the glomerular mesangium, profile miRNA expression in situ in the developing renal stroma, and proposes a second mechanism whereby stromal miRNAs are required for maintaining mesangial integrity during kidney development. Thus, using high-throughput miRNA profiling and transcriptome microarray analysis, 72 potential dysregulated miRNA:mRNA interactions were identified, which were enriched in transcripts known to regulate apoptosis. The ectopic expression of the proapoptotic protein, Bim, and elevated levels of downstream p53 effector genes (*Bax*, *Trp53inp1, Jun* and *Cdkn1a*) in *FoxD1*^*GC*^*;Dicer*^*fl/fl*^ kidneys support the idea that misregulated apoptosis contributes to the renal phenotype.

The renal stroma has been shown to play crucial roles in many aspects of kidney development, including modulation of nephron progenitor self-renewal and differentiation, as well as establishment of the cortico-medullary axis (Yu et al. 2009; Das et al. 2013). Although Nakagawa et al. (2015) reported decreased nephron progenitors in *FoxD1*^*GC*^*;Dicer*^*fl/fl*^ kidneys, we have several experimental lines of evidence that support an expansion of this population in our model, including immunofluorescence, microarray data, and quantitative real-time PCR for nephron progenitor-specific genes. Moreover, a consistent feature in stromal defect models is the expansion of nephron progenitors and aberrant nephrogenesis (Levinson et al. 2005; Das et al. 2013; Hum et al. 2014). Recent studies have demonstrated the cellular plasticity of the renal stroma to adopt a nephron progenitor cell fate at low frequency, prior to lineage restriction by E11.5 (Brunskill et al. 2014; Kobayashi et al. 2014). Although such cellular plasticity may contribute to the expansion of *FoxD1*^*GC*^*;Dicer*^*fl/fl*^ nephron progenitors, two lines of evidence argue against this possibility. Expansion of nephron progenitors in response to loss of stromal miRNAs occurs from E15.5 onward, a time point whereby lineage restriction of the renal compartments has been established. Moreover, fate mapping of the *FoxD1*^*GC*^*;Dicer*^*fl/fl*^ kidneys showed appropriate renal stroma patterning with no evidence for a cell fate switch. Instead, absence of stromal miRNAs appears to result in dysregulation of stromal Fat4 signaling with a resulting noncell autonomous signaling that is likely to mediate suppression of progenitor differentiation. Aside from the observed difference in nephron progenitors in our model compared to Nakagawa et al. (2015), our microarray analysis suggests no overt changes in Wnt signaling, which is in keeping with a morphologically normal-appearing renal papilla in our model. We speculate that background strain differences and/or mosacism in Cre excision may account for these differences.

The loss of stromal miRNAs also resulted in significant perturbations to normal vascular development and maintenance, which is in keeping with the inherent differentiation properties of the renal stroma. Thus, the reduced smooth muscle in afferent arterioles and decreased renin-expressing cells in *FoxD1*^*GC*^*;Dicer*^*fl/fl*^ kidneys reflect an intrinsic requirement for stromal miRNAs in these stromal derivatives. During glomerular development, *FoxD1*^*GC*^*;Dicer*^*fl/fl*^ kidneys exhibited progressive loss of the mesangium. Mesangial development is dependent on normal endothelial migration into the developing glomerulus, secretion of Pdgf*β* by the glomerular endothelium, the initial specification of mesangial cells, and migration of mesangial cells into the glomerular cleft, all of which occurred in *FoxD1*^*GC*^*;Dicer*^*fl/fl*^ kidneys. Interestingly, the *FoxD1*^*GC*^*;Dicer*^*fl/fl*^ mesangial phenotype arises most often in mature glomeruli during late-stage nephrogenesis, supporting the idea that stromal derived miRNAs are required for maintaining the integrity of the glomerular mesangium. Thus, the requirement for stromal miRNAs appears to be in the subsequent maintenance and survival of mesangial cells during development.

Mesangial loss can arise as a secondary pathological defect as a consequence from dysfunctional glomerular endothelial cells or podocytes (Morita et al. 1998). The *FoxD1*^*GC*^*;Dicer*^*fl/fl*^ model supports a mechanism whereby mesangium loss occurs primarily as a result of an intrinsic mesangial defect. However, a potential confounding factor is that *FoxD1*^*GC*^ is expressed in podocytes (Brunskill et al. 2011, 2014; Kobayashi et al. 2014), and impaired podocyte differentiation has been reported in this model (Nakagawa et al. 2015). However, several previous studies demonstrated that podocyte-specific loss of miRNAs results in an increase in mesangial markers, and an absence of glomerular aneurysms (Harvey et al. 2008; Ho et al. 2008; Shi et al. 2008). Together, the observations suggest that stromal miRNAs are required for mesangial survival and integrity of the mesangial network, and that their absence results in progressive mesangial cell loss.

Consistent with our previous studies, ectopic apoptosis occurs in the mesangium and interstitial cells with upregulation of the proapoptotic protein Bim. Indeed, several stromal miRNAs were identified that are predicted to suppress *Bim* (miRs-10a, 18a, 19b, 24, 30c, 92a, 106a, 130a, 152, 181a, 214, 222, 302a, 370, and 381). Of these, miRs-18a, 19b, 92a, 106a, and 222 have previously been validated experimentally in other studies based on Ingenuity Pathway analysis.

p53 functions in apoptosis as a direct transcriptional activator of proapoptotic genes including *Bax*, *Trp53inp1*, *Puma,* and *Apaf-1* (Moroni et al. 2001; Nakano and Vousden 2001; Okamura et al. 2001; Chipuk et al. 2004). Indeed, overexpression of these proapoptotic transcripts alone is sufficient to induce an apoptotic cascade (Pastorino et al. 1998; Perkins et al. 1998; Liu et al. 2005). Recent studies have also demonstrated the indispensable requirement for p53 signaling in both kidney development, as well as renal repair after ischemia–reperfusion injury (Saifudeen et al. 2009; Sutton et al. 2013; Ying et al. 2014). While *FoxD1*^*GC*^*;Dicer*^*fl/fl*^ kidneys display evidence for upregulation of p53-effector genes, p53 protein levels remain unchanged, suggesting that stromal miRNAs directly repress p53-effector genes. Of the 175 miRNAs identified in the renal stroma, 25 miRNAs are experimentally validated, or have been predicted with high confidence, to interact with p53-effector genes. miRNAs are known to target multiple genes in a single signaling pathway, or target genes of distinct pathways that converge into a common biological process (such as apoptosis) as a mean of synergizing their repressive effect (He and Hannon 2004; Ho and Kreidberg 2012). Thus, in addition to the upregulation of Bim that is common to several kidney specific-*Dicer* knockout models, the *FoxD1*^*GC*^*;Dicer*^*fl/fl*^ model supports modulation of the p53 pathway as an additional mechanism for *Dicer*-null models with elevated cell death.

In summary, stromal miRNAs are required for normal nephrogenesis and formation of the renal vascular network, in particular for glomerular mesangial maintenance. The loss of stromal miRNAs results in ectopic apoptosis in the mesangium and interstitial cells, progressing to mesangial loss and eventually glomerular microaneurysms. Mesangial defects are observed in several human mutations resulting in diffuse mesangial sclerosis and renal failure in early childhood (including *WT1*, *LAMB2,* and *phospholipase epsilon C1*) (Zenker et al. 2004; Hinkes et al. 2006; Schumacher et al. 2007), as well as chronic kidney disease associated with toxin-induced glomerulopathy, diabetic nephropathy, and thrombotic microangiopathies (Morita et al. 1998). Thus, loss of miRNA function in the glomerular mesangium may be involved in these pathological processes. Future development of inducible mesangial-specific transgenic mouse models will be useful in defining the function of miRNAs in the mesangium in adulthood.

## Supporting Information

**Table S1.** Primers for qPCR. **Table S2.** Primers for riboprobe synthesis. **Table S3.** miRNAs enriched in the renal stroma. miRNA *C*_T_ value was normalized to U6 RNA, and ranked according to their average Δ*C*_T_ value. Enriched miRNAs in the stroma are denoted by a smaller Δ*C*_T_ value and indicated in red. **Table S4.** Differentially expressed genes in E18.5 *FoxD1*^*GC*^*; Dicer*^*fl/fl*^ kidneys. **Table S5.** Differentially expressed genes in E15.5 *FoxD1*^*GC*^*; Dicer*^*fl/fl*^ kidneys. **Table S6.** Ingenuity pathway analysis of stromal miRNAs’ interactions with Bim-associated genes. **Table S7.** Ingenuity pathway analysis of stromal miRNAs’ interactions with p53-associated genes.
